# A Role for the Transcription Factor *Arid3a* in Mouse B2 Lymphocyte Expansion and Peritoneal B1a Generation

**DOI:** 10.3389/fimmu.2017.01387

**Published:** 2017-10-24

**Authors:** Katrin Habir, Shahin Aeinehband, Fredrik Wermeling, Stephen Malin

**Affiliations:** ^1^Department of Medicine, Center for Molecular Medicine, Karolinska University Hospital, Karolinska Institutet, Stockholm, Sweden

**Keywords:** *Arid3a*, B cell development, spleen, bone marrow, antibody formation

## Abstract

The initiation, commitment, and terminal differentiation of the B cell lineage is stringently controlled by the coordinated action of various transcription factors. Among these, *Arid3a* has previously been implicated in regulating early B lymphopoiesis, humoral immune responses to phosphocholine, and furthermore to promote the B1 over the B2 cell lineage. We have now interrogated the function of *Arid3a* in the adult mouse using conditional mutagenesis. We demonstrate that loss of *Arid3a* does not affect early B cell development or lineage commitment but rather loss of this transcription factor results in a broad expansion of bone marrow B lymphopoiesis in a manner that reflects its developmental expression pattern. Furthermore, loss of *Arid3a* resulted in expanded splenic B cell numbers with the exception of the B1 lineage that was maintained at normal numbers. However, B1a lymphoyctes were reduced in the peritoneal cavity. In addition, antibody responses to phosphocholine were attenuated in the absence of *Arid3a*. Hence, functional *Arid3a* is required in mature B cells for specific immune responses and for generating normal numbers of B cells in a subset dependent manner.

## Introduction

B lymphocytes form the humoral arm of adaptive immunity through their unique ability to construct and secrete antibodies. The B cell lineage can be broadly separated into the B1 and B2 lineages through the combination of surface markers, ontogeny, physiological location, and specificity for antigen (1). The microRNA *Let-7* and RNA-binding protein LIN28b have a critical role in specifying this B1 versus B2 lymphocyte lineage (2).

*Arid3a* was identified through its ability to bind and transcriptionally regulate the immunoglobulin heavy chain gene (3, 4) and has been recently claimed to be a critical downstream target of the *Let-7*/LIN28b axis (5). There have been conflicting reports on the function of *Arid3a* within the B cell lineage. Germline deletion of *Arid3a* leads to multiple defects in both hematopoietic progenitors and bone marrow resident B cells, including B1 cells (6). Additional approaches using knockdown or overexpression of *Arid3a* followed by transplantation into lymphopenic hosts has led to the hypothesis that *Arid3a* is required for specifying the B1 cell fate over the B2 cell fate (5). Conversely, lymphocyte-specific deletion of *Arid3a* through RAG-deficient blastocyst complementation experiments did not reveal any significant differences in mature B cell development (6). Similarly, transgenic overexpression of a dominant-negative ARID3A protein had no effect on bone marrow B cell development or on the B1 versus B2 lineage in the peritoneum (7).

To circumvent the embryonic lethality of *Arid3a* germline deleted mice, we have created a conditional loss-of-function allele and combined this with B cell-specific *Cre*-mediated deletion. Comprehensive analysis of the B cell compartment in these mice revealed expansion of the bone marrow pro-B, pre-B, immature and recirculating stages of B cell development upon *Arid3a* deletion, as well as defects in humoral immunity. In notable contrast, adult B1 cells are reduced, but only in the peritoneal cavity, following the loss of *Arid3a*.

## Results

### Conditional Loss of *Arid3a* and B Cell Development

**Figure 1:**
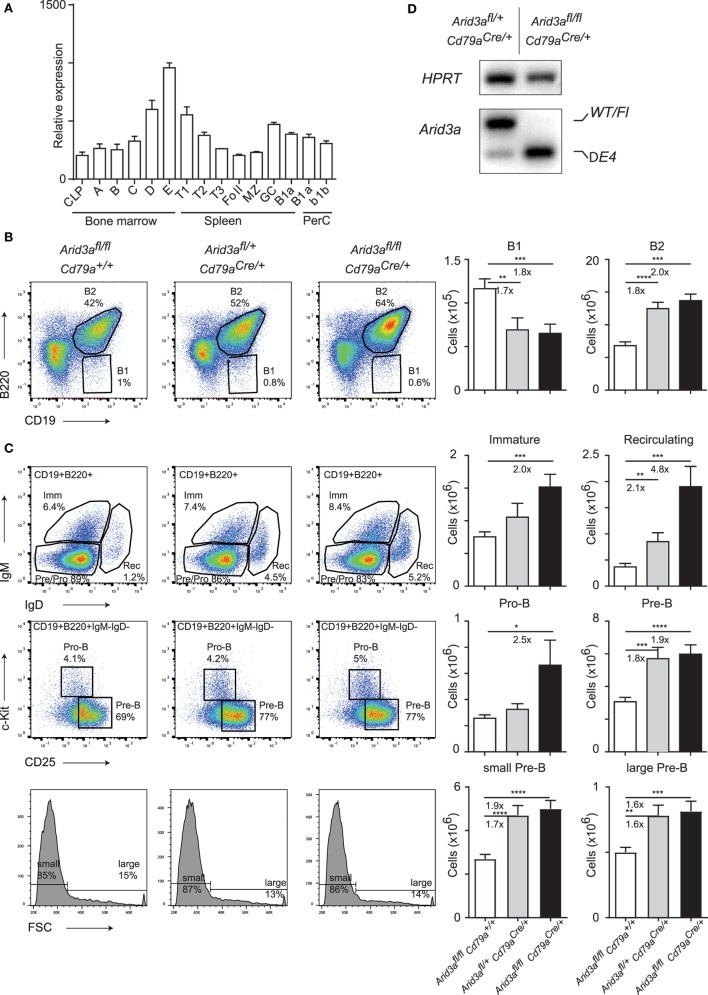
Conditional loss of *Arid3a* and B cell development. **(A)** Relative expression of *Arid3a* in B cell subsets in the bone marrow (common lymphoid progenitor and fractions A to E), spleen (transitional and mature stages), and peritoneal cavity using data from the ImmGen database. **(B,C)** Flow cytometry of bone marrow B cell development in *Arid3a^fl/fl^, Arid3a^fl/+^ Cd79a^Cre/+^*, and *Arid3a^fl/fl^ Cd79a^Cre/+^* mice. Flow cytometry plots are shown on the left. Absolute cell numbers for B1 (CD19^+^B220^lo^), B2 (CD19^+^B220^+^), immature B (CD19^+^B220^+^IgM^+^IgD^−^), recirculating B (CD19^+^B220^+^IgM^−^IgD^+^), pro-B (CD19^+^B220^+^IgM^−^IgD^−^ c-Kit^+^CD25^−^), and pre-B (CD19^+^B220^+^IgM^−^IgD^−^c-Kit^−^CD25^+^) cell populations are shown on the right. Error bars represent SEM and *n* = 17–24 for each group. *p*-Values were determined by Student’s *t*-test and fold changes are indicated. **(D)** Deletion of the conditional *Arid3a* allele determined by RT-polymerase chain reaction in sorted pro-B cells from the bone marrow of *Arid3a^fl/+^ Cd79a^Cre/+^* and *Arid3a^fl/fl^ Cd79a^Cre/+^* aged 4–6 weeks of age. The full length wild-type and floxed allele (WT/Fl) and exon 4 deleted allele (ΔE4) are indicated. *HPRT* was used as a loading control.

The expression pattern of the transcription factor *Arid3a* is believed to be largely restricted to the B cell lineage within the hematopoietic system (8). We first determined the pattern of *Arid3a* expression by examining the ImmGen database (9) and could confirm that *Arid3a* is restricted to B cells with the exception of prominent expression also within granulocytes. Surprisingly, *Arid3a* was not strongly expressed in hematopoietic stem cells or progenitors, but instead showed upregulation within B cell progenitors resident in the bone marrow (Figure 1A). Specifically, *Arid3a* expression was upregulated through successive maturation steps from the pro-B to immature B cell stages. Interestingly, B1 cells did show only moderately increased expression of *Arid3a*.

We next wished to interrogate the *in vivo* function of *Arid3a* by genetic loss-of-function analysis. We generated a floxed allele of *Arid3a* by first removing the *Lacz/Neo* cassette of *Arid3a^*tm1a(KOMP)Wtsi*^* by FLPE-mediated recombination to create an allele that contains *Loxp* sites flanking exon 4 (Figure S1A in Supplementary Material). This encodes for amino acids 237–261 that are crucial for *Arid3a* DNA binding (4), and additionally loss of exon 4 will result in an out of frame allele and a nonsense protein. We then crossed this floxed allele with the B cell-specific *Cre* line *Cd79a^Cre^* (*Mb1^Cre^*), which initiates deletion at the transition from the common lymphoid progenitor to the pro-B cell stage of development (10). We therefore created littermate cohorts of *Arid3a^fl/fl^* (herein referred to as control mice), *Arid3a^fl/+^Cd79a^Cre/+^* (herein referred to as heterozygous mice), and *Arid3a^fl/fl^Cd79a^Cre/+^* (herein referred to as homozygous mice) and analyzed the B cell compartment in the bone marrow of these mice at 4–6 weeks of age. We found that loss of *Arid3a* influenced the relative abundance of the B1 (defined B220^lo^CD19^+^) populations in the bone marrow, with approximate 1.7-fold decreases in B1 cells in heterozygous versus control mice (*p* = 0.0047) and 1.8-fold decreases in homozygous versus control mice (*p* = 0.0006). In contrast, the B2 population (defined B220^+^CD19^+^) was expanded 1.7-fold in heterozygous versus control (*p* < 0.0001) and 2-fold increased in the homozygous versus control cohorts (*p* = 0.0006) (Figure 1B). We next determined which stage of B cell development was responsible for this increase in B2 cells. The first committed pro-B stage of B cell development (defined B220^+^CD19^+^IgM^−^IgD^−^c-Kit^+^CD25^−^) was increased 2.5-fold in homozygous mice upon loss of *Arid3a* (*p* = 0.038). Later stages of B cell development were also expanded. The pre-B population (defined B220^+^CD19^+^IgM^−^IgD^−^c-Kit^−^CD25^+^) including the large pre-B and small and pre-B subpopulations increased approximately 1.9-fold in both heterozygous and homozygous mice (control versus heterozygous *p* = 0.0001: control versus homozygous *p* < 0.0001). Immature-B (defined B220^+^CD19^+^IgM^+^IgD^−^) were increased 2-fold in the homozygous versus control mice (*p* = 0.038), whereas recirculating B cells (defined B220^+^CD19^+^IgM^−^IgD^+^) demonstrated dosage-dependent increases in cell number upon loss of *Arid3a* ranging from 2.1-fold (control versus heterozygous *p* = 0.0043) to 4.8-fold (control versus homozygous *p* = 0.0002) (Figure 1C; Figure S1B in Supplementary Material), resulting in an increase in bone marrow cellularity (Figure S1C in Supplementary Material).

*Cd79a^Cre^* is well established as an efficient deleter of floxed alleles in B cells. We further confirmed this by sorting pro-B cells by fluorescence-activated cell sorting and assessing deletion of *Arid3a* at the *mRNA* level. Cells from heterozygous mice contained both wild-type and exon-4 deleted *Arid3a*, whereas cells sorted from homozygous mice only contained the deleted allele (Figure 1D). We further sequenced this deleted allele and confirmed that an out of frame mRNA is produced upon loss of exon 4 resulting in a protein with multiple premature stop codons (Figure S1D in Supplementary Material). In summary, loss of *Arid3a* leads to reductions in bone marrow B1 cell numbers and conversely a more noticeable expansion of all stages of B2 cell development.

### Expanded Peripheral B2 Cell Populations in *Arid3a*-Deficient Mice

**Figure 2:**
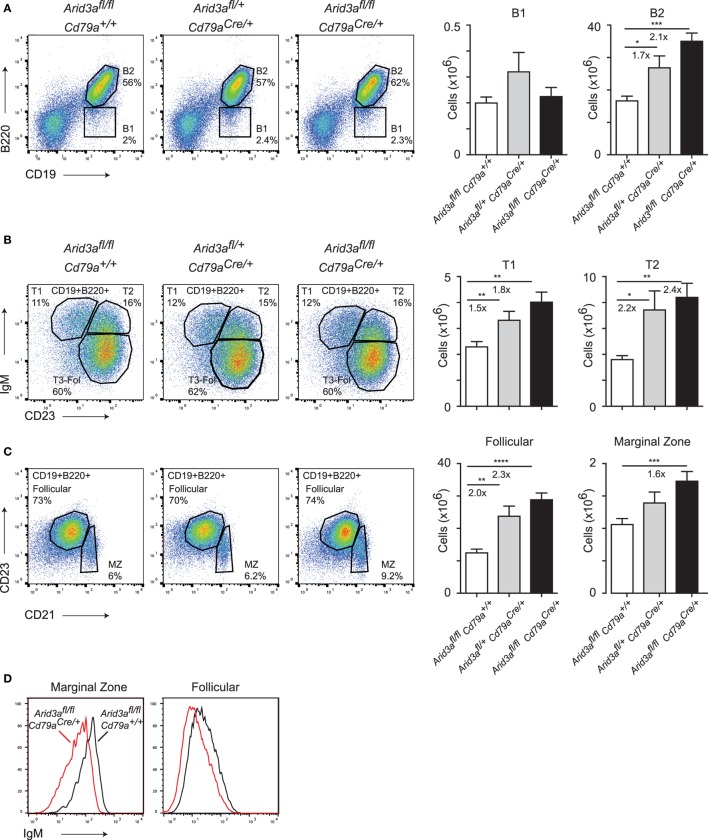
The *in vivo* function of *Arid3a* in splenic B cell development. **(A)** Flow cytometry of splenic B1 cells (CD19^+^B220^lo^) and B2 cells (CD19^+^B220^+^) in the left panel with quantification shown on the right for the indicated genotypes from mice aged 10–12 weeks. **(B)** Analysis of Transitional 1 (CD19^+^B220^+^IgM^+^CD23^−^) and Transitional 2 (CD19^+^B220^+^IgM^+^CD23^+^) B cells. **(C)** Follicular B (CD19^+^B220^+^CD21^lo^CD23^+^) and marginal zone B (CD19^+^B220^+^CD21^hi^ CD23^lo^) cell populations in the spleen. Error bars represent SEM and *n* = 9–32 for each group. *p*-Values were determined by Student’s *t*-test and fold changes are indicated. **(D)** Expression of IgM on marginal zone and follicular B cells.

The expansion of B cell numbers in the bone marrow could result in further abnormalities in peripheral B cell numbers. We analyzed control, heterozygous, and homozygous mice at 10–12 weeks of age and quantified the absolute cell numbers of various B cell subsets in the spleen. We observed a twofold expansion (*p* = 0.0001) in B2 cell numbers in homozygous versus control mice, but no difference in B1 cell numbers. This increase in B2 cells was dosage dependent as loss of one *Arid3a* allele also resulted in increases in absolute numbers of B cells (*p* = 0.0032) (Figure 2A; Figure S2A in Supplementary Material), with subsequent increases in spleen cellularity and CD3^+^ cell numbers (Figures S2B,C in Supplementary Material). We next wished to determine which subset was responsible for the increase in B2 cell number. Transitional 1 B cells (B220^+^CD19^+^IgM^+^CD23^−^) were increased in both heterozygous (*p* = 0.0024) and homozygous (*p* = 0.0027) versus control mice. Transitional 2 B cells (B220^+^CD19^+^IgM^+^CD23^+^) were increased up to 2.4-fold (*p* = 0.0036) in homozygous mice versus controls (Figure 2B). An alternative gating strategy incorporating CD93 also revealed increases in immature splenic populations (Figure S2D in Supplementary Material). Similarly, the number of follicular B cells (defined B220^+^CD21^+^CD21^lo^CD23^+^) was also increased in cell numbers and again appeared to be dosage sensitive to loss of *Arid3a* (control versus heterozygous *p* = 0.0022: control versus homozygous *p* < 0.0001). Marginal zone B cells (defined B220^+^CD21^+^CD21^hi^CD23^lo^) were also increased in homozygous versus control mice (*p* = 0.0095) (Figure 2C). Interestingly, surface IgM levels were decreased on follicular and marginal zone B cells in homozygous mice (Figure 2D). In summary, peripheral B cell numbers are expanded upon loss of *Arid3a* in similar fashion to B cell progenitors in the bone marrow.

### The *In Vivo* Role of *Arid3a* in the B1 versus B2 Cell Development

**Figure 3:**
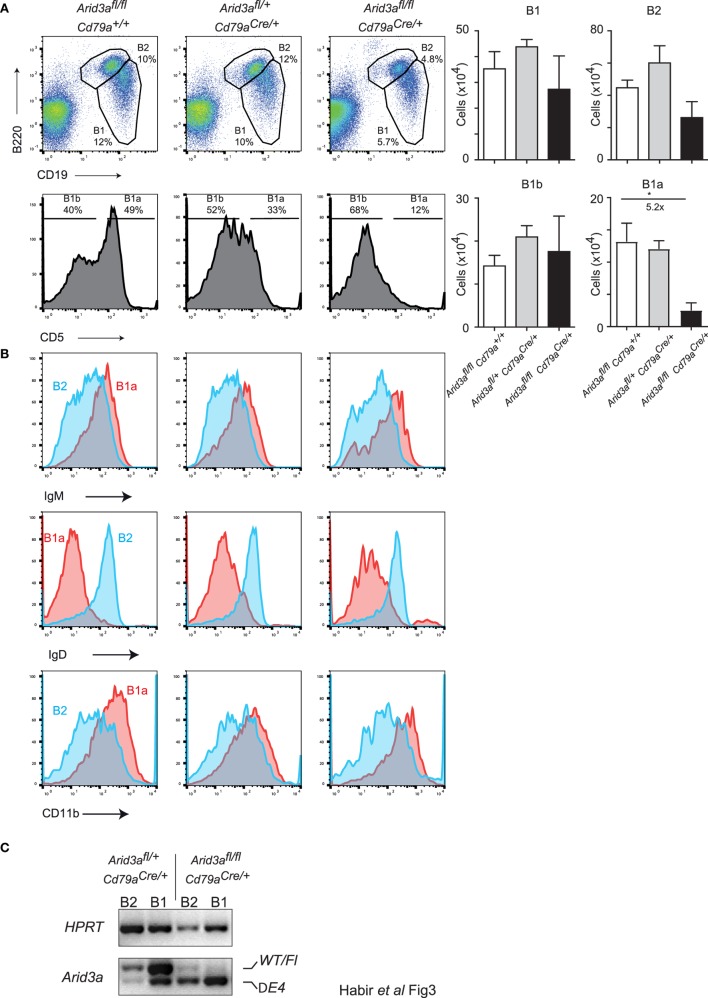
*Arid3a* is required for normal peritoneal cavity B1a cell development. **(A)** Flow cytometry of B1 cells (CD19^+^B220^lo^) and B2 cells (CD19^+^B220^+^) taken from peritoneal lavages of mice aged 10–12 weeks old. B1 cells were further gated for CD5 to determine B1a (CD19^+^B220^lo^CD5^+^) and B1b (CD19^+^B220^lo^CD5^−^) populations shown in the bottom panel. Error bars represent SEM and *n* = 3–8 for each group. *p*-Values were determined by Student’s *t*-test and fold changes are indicated. **(B)** Cell surface phenotype of B1a (CD19^+^B220^lo^CD5^+^) and B2 (CD19^+^B220^+^) cells. **(C)** Deletion of the conditional *Arid3a* allele was shown by RT-polymerase chain reaction in B1 and B2 cells sorted from peritoneal cavity in *Arid3a^fl/+^ Cd79a^Cre/+^* and *Arid3a^fl/fl^ Cd79a^Cre/+^* mice, 10–12 weeks of age. The full length wild-type and floxed allele (WT/Fl) and exon 4 deleted allele (ΔE4) are indicated. *HPRT* was used as a loading control.

*Arid3a* has recently been positioned as a key regulator of the B1 versus B2 lineage (5, 11). The reduction of B1 cells in the bone marrow indicated that this phenotype is also present upon conditional loss of *Arid3a* in the adult mouse. We therefore analyzed a key reservoir of B1 cells in the mouse, namely the peritoneal cavity. We observed a strong decrease in the relative abundance of B1a (CD19^+^B220^lo^CD5a^+^) cells in homozygous mice, being 5.2-fold (*p* = 0.0306) reduced relative to controls. B2 cells and B1b (CD19^+^B220^lo^CD5a^−^) cells were not significantly reduced in numbers (Figure 3A). These B1a cells were further confirmed to have a cell surface phenotype of IgM^hi^, IgD^lo^, and CD11b^+^ (Figure 3B). An alternative gating strategy to define B1 cells (CD19^+^CD11b^+^) produced similar results (Figures S3A,B in Supplementary Material). Finally, we confirmed that peritoneal lymphocytes were deleted for *Arid3a* by sorting B1 and B2 cells and determining *Arid3a* deletion. Similar to the results from bone marrow pro-B cells, homozygous mice displayed efficient deletion of exon 4 of *Arid3a* (Figure 3C). We could therefore confirm that *Arid3a* is required for normal peritoneal B1a cell development.

### The Function of *Arid3a* in Humoral Immunity

**Figure 4:**
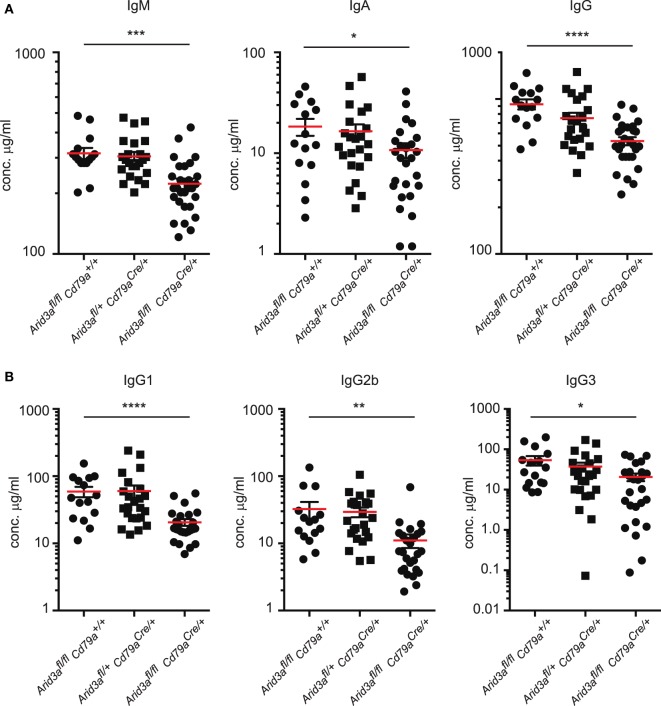
A requirement for *Arid3a* in humoral immunity. **(A)** Plasma was collected from naïve mice of the indicated genotypes aged 10–12 weeks old and analyzed for total IgM, IgA, and IgG levels with Enzyme-Linked ImmunoSorbent Assay. **(B)** IgG1, IgG2b, and IgG3 antibody titers. *n* = 15–26 for each group and *p*-values were determined by Student’s *t*-test.

The humoral immune response of mouse B cells has been reported to be influenced by the transcription factor *Arid3a*. We measured total plasma immunoglobulin levels in naïve mice and found significant decreases in IgM (*p* = 0.0003), IgA (*p* = 0.039), and IgG (*p* = 0.0001) in homozygous mice compared to controls (Figure 4A), similar to that reported in mice expressing dominant-negative *Arid3a* (7) and the very rare survivor mice from the germline deleted *Arid3a* strain (6). Within the IgG fraction, levels of IgG1, IgG2b, and IgG3 were all significantly reduced (Figure 4B). We did not observe any significant differences in heterozygous mice relative to control mice.

**Figure 5:**
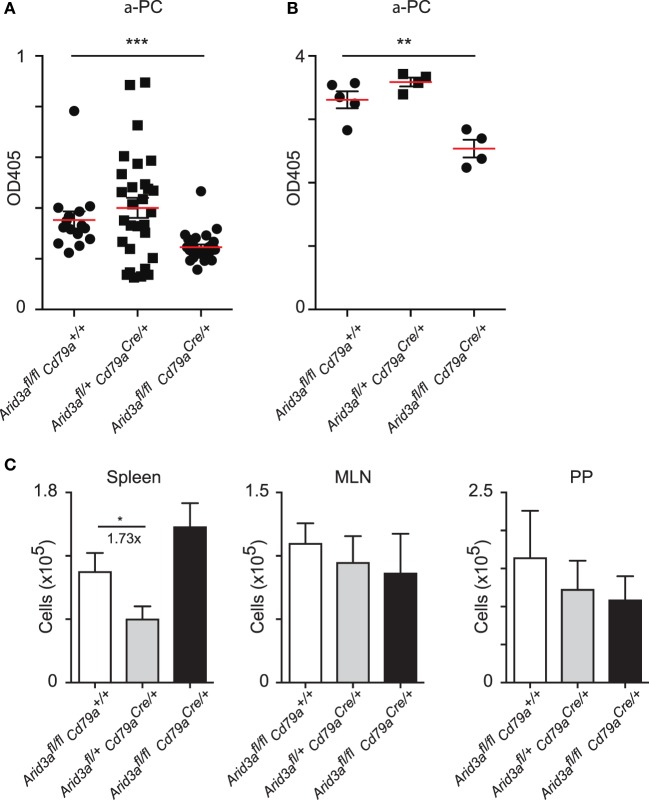
Normal antibody production against phosphocholine requires *Arid3a*. **(A)** Enzyme-Linked ImmunoSorbent Assay measurements of anti-phosphocholine IgM in naïve mice from the indicated genotypes *n* = 15–28 for each group. **(B)** Anti-phosphocholine IgM levels 14 days after PC-KLH immunization *n* = 4–5 for each group. **(C)** Absolute number of cells with a germinal center phenotype (CD19^+^B220^+^IgD^−^GL7^+^CD95^+^) in spleen, Peyer’s patches, and mesenteric lymph nodes after immunization with PC-KLH/Alum. *n* = 4–6 for each group. *p*-Values determined by Student’s *t*-test.

A consistent feature of mice with perturbed *Arid3a* function is reduced antibody titers against the hapten phosphocholine, which in C57/Bl6 mice is partially dependent on V(D)J rearrangements that have incorporated *V_H_S107.1.42* (12, 13). We assayed titers of IgM against phosphocholine and observed significant decreases in naïve homozygous mice versus that of controls (*p* = 0.0006) (Figure 5A). We next immunized mice with the antigen phosphocholine linked to Keyhole limpet hemocyanin (PC-KLH) emulsified in Alum to study germinal centre formation and specific T cell-dependent immune responses. We observed a reduction in anti-phosphocholine IgM responses 14 days after immunization (*p* = 0.0056) (Figure 5B). We next tested if this reduced anti-PC response was due to an inability to form germinal centers. Activated B cells (CD19^+^B220^+^IgD^lo^CD95^+^GL7^+^) could be readily detected in the spleen following immunization in all genotypes and were also present in normal numbers in the Peyer’s patches and mesenteric lymph nodes regardless of the *Arid3a* genotype (Figure 5C).

## Discussion

The regulation of the B1 versus the B2 cell fate is thought to be controlled either through antigen receptor specificity within the immature and transitional B cell stages, as ontogenically separate lineages, or a combination of these two models (14). Several transcription factors have been shown to effect the relative abundance of B1 versus B2 cells in secondary lymphoid organs, including *Ebf1* (15), classical NF-kappaB signaling (16), and most recently *Bhlhe41* (17). *Arid3a* has also been proposed to critically regulate the B1 versus B2 cell fate (11). Using conditional mutagenesis in the adult mouse, we found no evidence that expression of *Arid3a* is required for the production of B1 lymphocytes at the expense of B2 cells in the spleen, in agreement with studies that used RAG-deficient blastocyst complementation with *Arid3a* germline deleted ES cells (6) and overexpression of dominant-negative form of *Arid3a* (7). However, we did observe a loss of B1a cells in the peritoneal cavity, indicating that *Arid3a* has a very specific function in the generation of peritoneal B1a lymphocytes and is possibly responsible for the reduction in the IgM titers we observed. One possibility is that *Arid3a* may regulate migration of B1a cells to the peritoneal cavity.

Loss of *Arid3a* resulted in the expansion of all the B cell developmental stages in the bone marrow. This stage specific affect mirrors the expression pattern of *Arid3a*. Previous analysis of the less than l% of germline deleted *Arid3a* mice that survived to adulthood had indicated a possible role for this transcription factor in early B cell development (6); however, this germline-deficient strain has strong reductions in the common lymphoid progenitor compartment. Mature B cells were generated normally from *Arid3a*^−^*^/^*^−^ when using RAG-deficient blastocyst complementation, indicating that bone marrow B cell development had proceeded normally (6). Hence, the loss of B cell progenitors in *Arid3a*^−^*^/^*^−^ is probably due to the profound developmental defects in hematopoietic progenitors that occur in this strain. Similar results were obtained by conditional deletion of the related *Arid3b* in the mouse, which also resulted in a reduction of common lymphoid progenitors (18). Interestingly, follicular B cells were also expanded in this strain. It is possible that Arid3a and Arid3b have redundant functions during B lymphopoiesis, and combined deletion of these two factors will be required to confirm this.

Although we could demonstrate that the *mRNA* from our deleted allele should result in a missense protein, we were not able to measure ARID3A protein levels in our experiments through western blot experiments despite using multiple commercially available antibodies. We cannot therefore formally rule out that a truncated protein is produced through evasion of nonsense-mediated decay. However, we note that loss of exon 4 in the highly related *Arid3b* deleted locus resulted in loss of this protein (18).

Heterozygous or homozygous loss of Arid3a resulted in a complex set of phenotypes. Within bone marrow B cell development, heterozygous animals had similar increases in pre-B cells as homozygous mice, whereas for pro-B, immature and recirculating cells, loss of both alleles produced a more prominent expansion. This dosage pattern was conserved in spleen populations. This expansion of B cell progenitors we observed may have implications for B cell acute lymphoblastic leukemia (B-ALL). Knockdown of ARID3A in 70Z/3 and Ba/F3 B cell progenitor cell lines resulted in increased proliferation, and *ARID3A* was downregulated in a subset of B-All patients (19). Our results would be consistent with *Arid3a* suppressing proliferation in B cell progenitors. Alternatively, *Arid3a* may also have a function in cell survival. Regardless, expansion of certain B-ALL clones may be sensitive to the dosage of ARID3A, as normal B lymphopoiesis is expanded upon loss of one or two *Arid3a* alleles.

Loss of *Arid3a* results in specific defects in humoral responses, especially a partial defect in IgM responses against phosphocholine. The defects observed in immature bone marrow B cell development in *Arid3a*-deficient B cells may partially be the consequence of a positive feedback loop, as mice that cannot secrete IgM have increased immature B cells (20). Hence, in *Arid3a*-deleted B cells, there is diminished production of phosphocholine binding natural IgM. This is probably resulting from reduced *V_H_S107.1.42* expression, which is regulated by ARID3A (6). In contrast to bone marrow progenitors, however, the reduction in peritoneal B1a cells and plasma immunoglobulin levels was only observed in homozygous mice.

Although the defects we observed upon loss of *Arid3a* may appear subtle, increased ARID3A expression in human B cells correlates with the severity of systemic lupus erythematosus (21), which may be indicative of a more general function of this transcription factor in autoimmunity. The reduction in “natural” IgM against phosphocholine could make the *Arid3a^fl/fl^Cd79a^Cre/+^* strain useful in disease models where these natural antibodies have been implicated, for example, in SLE models and atherosclerosis.

## Materials and Methods

### Mice

The *Arid3a^*tm1a(KOMP)Wtsi*^* allele was imported from the KOMP resource and *in vitro* fertilized with sperm from the B6.Cg-Tg(ACTFLPe)9205Dym/J strain to remove the *Frt* flanked selection and reporter cassette to create *Arid3a^fl/+^* mice. Mice were then further crossed with the *Cd79a^Cre/+^* strain to create control and homozygous mice. All experiments used mice at 4–6 or 10–12 weeks of age and were approved by the Stockholm Regional Board for Animal Ethics, permit numbers N46/14, N164/14, and N111/11. Mice were regularly controlled by the Swedish Veterinary Authorities.

### Genotyping with Polymerase Chain Reaction (PCR)

Polymerase chain reaction was performed after DNA extraction from ear biopsies of mice. The *Arid3a^fl/fl^* and Arid3a*^fl/+^* DNA fragments were amplified with the primers: 5′-CTCCTTCCTCTTGTCTCCTGTGTGG-3′ (ttR) and 5′-TGCCTGTTGGAAGGATGATCTGG-3′ (F); and with the primers 5′-GAGATGGCGCAACGCAATTAATG-3′ (loxF) and 5′-GATAGCTCAGGAGCTTCCTCACACC-3′ (R). The *Cd79a^Cre/+^* fragment was amplified with the following primers: 5′-CCTTGCGAGGTCAGGGAGCC-3′ (WT reverse), 5′-GTCCTGGCATCTGTCAGAG-3′ (Cre reverse) and 5′-GGCTCTGACCCATCTGTCTC-3′ (common forward). Primers ttR and F amplified a 354-bp wild-type fragment and a 470-bp postFlp fragment. Primers loxF and R amplified a 253-bp floxed fragment. Primers WT reverse and common forward gave a 477-bp wild-type fragment, and primers Cre reverse and common forward gave a 500-bp *Cd79a^Cre/^*specific product. PCR products were subsequently separated by electrophoresis through agarose gels and visualized by GelRed nucleic acid gel stain (Biotium).

### Flow Cytometry

Bone marrow from Femur and Tibia of the two hind legs of 4–6 weeks old mice, and spleen and peritoneal cavity from 10 to 12 weeks old mice was isolated. Tissues were then fractioned to single-cell suspensions and filtered through 100-µm cell strainers (Sigma-Aldrich) with phosphate-buffered saline (PBS) containing 0.5% fetal bovine serum (FBS). Erythrocytes were thereafter lysed with Ammonium-Chloride-Potassium Lysing Buffer. Single-cell suspensions of bone marrow, spleen, and peritoneal cavity were pre-incubated at a density of 100 × 10^6^ cells/ml with Fc Block (BD Biosciences), and cells were subsequently analyzed by a CyAn ADP (Beckman Coulter) flow cytometer with the following antibodies B220 (APC-Cy7 clone: RA3-6B2), anti-CD19 (Alexa Fluor 647 clone: eBio1D3), anti-CD25 (PE-Cy7 clone: PC61.5), anti-c-kit (PE clone: 2B8), anti-CD5 (PE clone: 53-7.3), anti-GL7 (APC clone: GL-7), anti-CD95 (PE-Cy7 clone: Jo2), anti-IgD (PerCP clone: 11-26c.2a), anti-IgM (Pacific Blue clone: Il/41), anti-CD23 (PE-Cy7 clone: B3B4), anti-CD21 (FITC clone: 7G6), and anti-CD11b (PerCPclone: M1/70). Immune cell populations were defined as follows: B-1 (CD19^+^B220^lo^) or (CD19^+^CD11b^+^), B-1a (CD19^+^B220loCD5^+^) or (CD19^+^CD11b^+^CD5^+^), B-1b (CD19^+^B220^lo^CD5^−^) or (CD19^+^CD11b^+^CD5^−^), B-2 (CD19^+^B220^+^), pro-B (CD19^+^B220^+^IgM^−^IgD^−^c-Kit^+^CD25^−^), pre-B (CD19^+^B220^+^IgM^−^IgD^−^c-Kit^−^CD25^+^), immature-B cells (CD19^+^B220^+^IgM^+^IgD^−^) recirculating-B cells (CD19^+^B220^+^IgM^−^IgD^+^), Transitional-1 (CD19^+^B220^+^IgM^+^CD23^−^) or (CD19^+^B220^+^CD93^+^IgM^+^CD23^−^), Transitional-2 (CD19^+^B220^+^IgM^+^CD23^+^) or (CD19^+^B220^+^CD93^+^IgM^+^CD23^+^), marginal zone B cells (CD19^+^B220^+^CD21^hi^CD23^lo^), follicular B cells (CD19^+^B220^+^CD21^int^CD23^hi^) germinal center B cells (CD19^+^B220^+^IgD^−^GL7^+^CD95^+^). Absolute cell numbers were calculated by first counting live cells using a BioRad TC-20 cell counter based on trypan blue exclusion. Cells within a defined gated quadrant were divided by the cells contained in a large gate encompassing all live cells and excluding debris as determined by forward and side scatter properties from the flow cytometry. This ratio was then multiplied by the cell count to give the absolute cell number.

### Cell Sorting and Deletion Analysis

Pro-B cells from bone marrow and B1 and B2 peritoneal cavity cells of *Arid3a^fl/+^Cd79a^Cre/+^* and *Arid3a^fl/fl^Cd79a^Cre/+^* mice were sorted into PBS containing 0.5% FBS before RNA extraction with the RNeasy Mini Kit (Qiagen). cDNA was synthesized with High capacity RNA-to-cDNA Kit (Applied Biosystems) followed by PCR with primers with the following Arid3a oligonucleotides: 5′-TGGACCTTTGAGGAGCAGTT-3′ (Primer 1) and 5′-GATGGAGGTAGGCAGGTTGA-3′ (Primer 2). A 255-bp PCR product was amplified from the floxed Arid3a allele and a 182-bp product from the deleted Arid3a allele. The Hypoxanthine guanine phosphoribosyl transferase (*HPRT*) gene was used as a control with primers GGGGGCTATAAGTTCTTTGC and TCCAACACTTCGAGAGGTCC generating a 312-bp PCR product. PCR products were then separated by electrophoresis through agarose gels and visualized by GelRed nucleic acid gel stain.

### Immunization and Enzyme-Linked ImmunoSorbent Assay (ELISA)

For immunization with PC-KLH (Biosearch Technologies Inc.), 100 µg of PC-KLH was precipitated on Alum and subsequently injected intraperitoneally in mice. Spleens were analyzed by flow cytometry 14 days after immunization. Specific IgM responses against PC-KLH was determined by ELISA using plates coated with PC-BSA (Biosearch Technologies), alkaline phosphatase coupled goat anti-mouse IgM (Southern Biotechnology Associates), and alkaline phosphatase yellow Liquid Substrate (Sigma-Aldrich). Purified rat anti-mouse IgG (H + L) (Sigma-Aldrich) was used as capture antibody to measure total plasma IgM, IgA, and IgG levels. AP-labeled goat anti-mouse IgM, IgA and IgG, IgG1, IgG2b, and IgG3 (Sigma-Aldrich) was used for detection.

### Statistical Analysis

Data were analyzed using GraphPad Prism. *p*-Values of <0.05 from Student’s *t*-tests were reported.

## Ethics Statement

All experiments were approved by the Stockholm Regional Board for Animal Ethics, permit numbers N46/14, N164/14, and N111/11. Mice were regularly controlled by the Swedish Veterinary Authorities.

## Author Contributions

KH performed most of the experiments. SA assisted with ELISA measurements. FW provided mouse lines and contributed to the planning of the experiments. SM devised the study, interpreted data and wrote the manuscript with contributions from all authors.

## Conflict of Interest Statement

The authors declare that the research was conducted in the absence of any commercial or financial relationships that could be construed as a potential conflict of interest.

## Supplementary Material

Figure S1 Schematic of the targeted *Arid3a* locus and bone marrow flow cytometry. **(A)** The *Frt* flanked *Neo/LacZ* cassette was removed through FLPE-mediated excision to create an allele that contains LoxP sites flanking exon 4. Exon 4 partially encodes for the DNA-binding domain and loss of this exon results in an out of frame allele. **(B)** Expanded bone marrow flow cytometry gating strategy. **(C)** Absolute numbers of live bone marrow cells. Error bars represent SEM and *n* = 23–36 for each group. *p*-Values were determined by Student’s *t*-test and fold changes are indicated. **(D)** Sequence of the wild-type and deleted *Arid3a* locus across exons 3–5 with the translated sequence underneath. The nonsense protein predicted from the exon 4 deleted *Arid3a* was confirmed by sequencing of the *mRNA*.

Figure S2 Expanded flow cytometry analysis of the spleen. **(A)** An example of an expanded flow cytometry gating strategy for splenic B cells, indicating lymphocyte and large gates. **(B)** Absolute numbers of live splenic cells. Error bars represent SEM and *n* = 9–33 for each group. *p*-Values were determined by Student’s *t*-test and fold changes are indicated. **(C)** Absolute numbers of splenic CD3^+^ cells. *n* = 8–15 for each group. **(D)** Absolute cell numbers of transitional B cells gated as Immature (CD19^+^B220^+^CD93^+^), T1 (CD19^+^B220^+^CD93^+^IgM^+^CD23^−^), T2 (CD19^+^B220^+^CD93^+^IgM^+^CD23^+^), and T3 (CD19^+^B220^+^CD93^+^IgM^lo^CD23^+^). *n* = 9–15 for each group.

Figure S3 Alternative gating analysis for peritoneal cavity B1 cells. **(A)** Flow cytometry of B1 cells (CD19^+^CD11b^+^) taken from peritoneal lavages of mice aged 10–12 weeks old, gated for CD5, to determine B1a (CD19^+^CD11b^+^CD5^+^) and B1b (CD19^+^B220^lo^CD5^−^) populations. Error bars represent SEM and *n* = 3–8 for each group. *p*-Values were determined by Student’s *t*-test and fold changes are indicated. **(B)** Cell surface phenotype of B1a (CD19^+^CD11b^+^CD5^+^) and B2 cells (CD19^+^CD11b^+^).
