# Supplementary figures and images for: A Role for the Transcription Factor Arid3a in Mouse B2 Lymphocyte Expansion and Peritoneal B1a Generation
Source: Front Immunol. 2017 Oct 24;8:1387. doi: 10.3389/fimmu.2017.01387 (PMC5660704; doi:10.3389/fimmu.2017.01387)

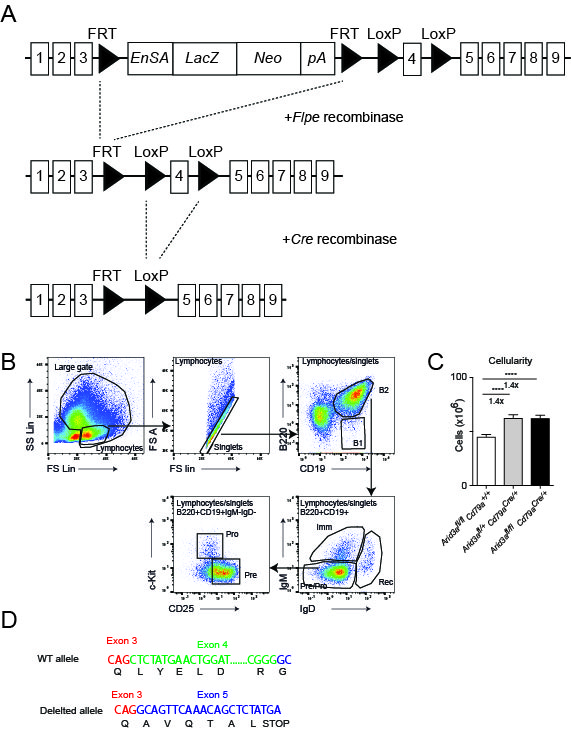

Supplement: Figure S1 — Schematic of the targeted Arid3a locus and bone marrow flow cytometry. (A) The Frt flanked Neo/LacZ cassette was removed through FLPE-mediated excision to create an allele that contains LoxP sites flanking exon 4. Exon 4 partially encodes for the DNA-binding domain and loss of this exon results in an out of frame allele. (B) Expanded bone marrow flow cytometry gating strategy. (C) Absolute numbers of live bone marrow cells. Error bars represent SEM and n = 23–36 for each group. p-Values were determined by Student’s t-test and fold changes are indicated. (D) Sequence of the wild-type and deleted Arid3a locus across exons 3–5 with the translated sequence underneath. The nonsense protein predicted from the exon 4 deleted Arid3a was confirmed by sequencing of the mRNA. [file image_1.jpeg]

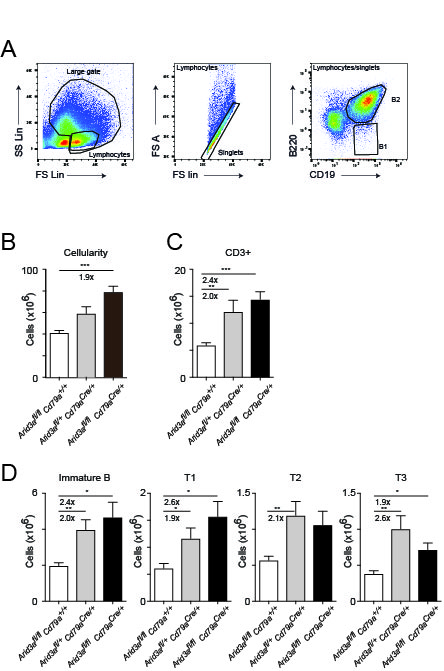

Supplement: Figure S2 — Expanded flow cytometry analysis of the spleen. (A) An example of an expanded flow cytometry gating strategy for splenic B cells, indicating lymphocyte and large gates. (B) Absolute numbers of live splenic cells. Error bars represent SEM and n = 9–33 for each group. p-Values were determined by Student’s t-test and fold changes are indicated. (C) Absolute numbers of splenic CD3+ cells. n = 8–15 for each group. (D) Absolute cell numbers of transitional B cells gated as Immature (CD19+B220+CD93+), T1 (CD19+B220+CD93+IgM+CD23−), T2 (CD19+B220+CD93+IgM+CD23+), and T3 (CD19+B220+CD93+IgMloCD23+). n = 9–15 for each group. [file image_2.jpeg]

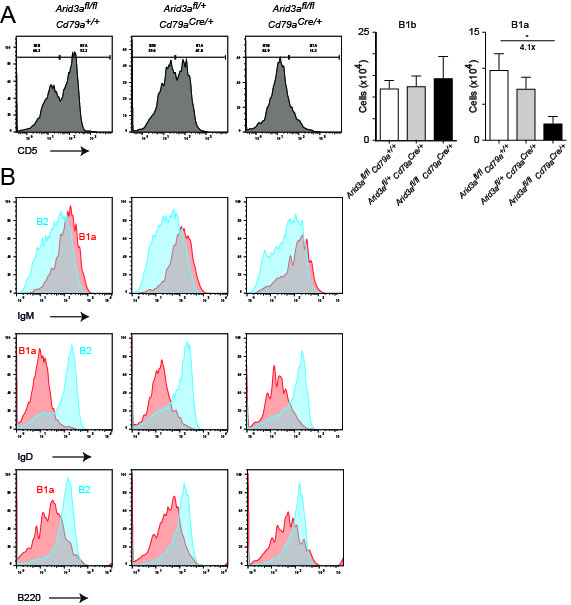

Supplement: Figure S3 — Alternative gating analysis for peritoneal cavity B1 cells. (A) Flow cytometry of B1 cells (CD19+CD11b+) taken from peritoneal lavages of mice aged 10–12 weeks old, gated for CD5, to determine B1a (CD19+CD11b+CD5+) and B1b (CD19+B220loCD5−) populations. Error bars represent SEM and n = 3–8 for each group. p-Values were determined by Student’s t-test and fold changes are indicated. (B) Cell surface phenotype of B1a (CD19+CD11b+CD5+) and B2 cells (CD19+CD11b+). [file image_3.jpeg]
